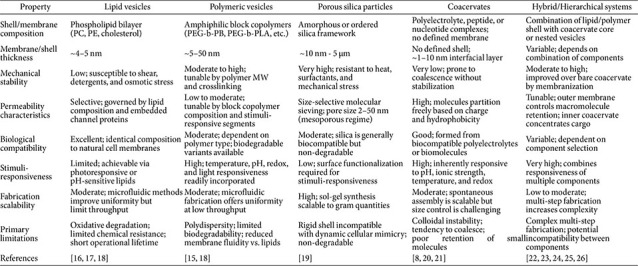# Bottom-Up Synthetic Biology for Artificial Cell Design: From Scaffold Materials to Functional Integration

**DOI:** 10.4014/jmb.2604.04021

**Published:** 2026-05-25

**Authors:** So-Hee Park, Seong-Min Jo

**Affiliations:** Department of Biomaterials Science, Pusan National University, Miryang 50463, Republic of Korea

**Keywords:** Artificial cells, Bottom-up synthetic biology, Origin-of-life, Biocatalysis

## Abstract

Cells are extraordinary biochemical systems that have evolved over billions of years into highly sophisticated units of life. Bottom-up synthetic biology seeks to reconstruct cell-like systems from non-living molecular components, producing artificial cells that capture essential cellular features while bypassing the complexity and fragility of living organisms. This approach offers a unique perspective on the organizational principles underlying cellular life and provides a platform for diverse biotechnological applications. In this review, we survey recent advances in bottom-up artificial cell design, covering five principal scaffold materials including the newly prominent coacervate-based and hybrid hierarchical compartments, and four canonical functional categories: cascade metabolism, protein synthesis, division, and energy production. We further discuss the expanding application landscape spanning industrial biocatalysis, therapeutic protein delivery, biosensing, and origins of life research. Finally, we critically evaluate the key technical limitations currently facing the field, including module compatibility, operational stability, and regulatory challenges, and outline the directions that must be pursued to advance artificial cells toward practical realization.

## Introduction

The cell is the fundamental unit of all living organisms, a microscale system of extraordinary biochemical sophistication that has evolved over approximately 4.0 billion years [1]. Within a volume of only a few picoliters, thousands of biochemical reactions are orchestrated in a spatiotemporally precise manner, enabling self-maintenance, energy transduction, information storage and retrieval, and ultimately self-reproduction. The question of whether such a system can be deliberately reconstructed from its molecular constituents remains one of the most profound challenges in modern science.

**Fig. 1:**
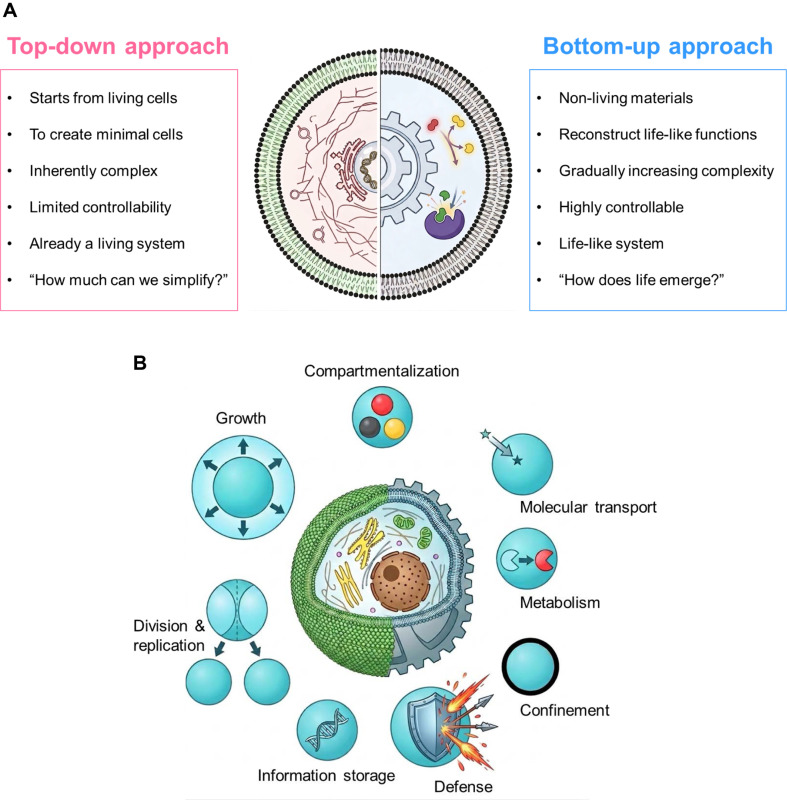
Schematic comparison of top-down and bottom-up approaches in synthetic biology. (**A**) The top-down strategy simplifies existing living cells, while the bottom-up strategy assembles cell-like systems from non-living molecular components through rational design and modular integration. (**B**) Key features of living cells that serve as design targets for artificial cell construction, including confinement, compartmentalization, metabolism, molecular transport, replication and division, defense, information storage, and growth.

Synthetic biology has emerged as a powerful discipline at the interface of biology, chemistry, and engineering, aiming to design novel biological systems with programmable functions [2]. Two complementary strategies define the field (Fig. 1A). The top-down approach begins with an existing living cell and systematically reduces or rewires its genetic content, as exemplified by the construction of a minimal synthetic genome in Mycoplasma mycoides [3]. In contrast, the bottom-up approach, which is the focus of this review, begins from non-living molecular components and seeks to assemble increasingly complex, cell-like systems through rational design and modular integration [4]. This perspective provides a unique insight into the organizational principles and origins of the cells.

Living cells, despite their biochemical prowess, present significant limitations when deployed as technological tools. They are metabolically expensive to maintain, sensitive to environmental perturbations, and difficult to engineer for a single biochemical function without interference from the broader cellular context [5]. These limitations have driven the development of artificial cells, engineered cell mimics that capture essential cellular features while avoiding inherent biological complexity. Although artificial cells lack many attributes of natural cells, their simplified biochemical composition may reflect characteristics of early primitive cells, offering a window into the fundamental principles that governed the emergence of cellular life.

The field has undergone a remarkable transformation in recent years, moving from early proof-of-concept demonstrations toward the assembly of increasingly integrated synthetic systems. Key advances include the reconstitution of energy transduction within polymersome membranes [6], cell-free gene expression within giant unilamellar vesicles (GUVs) [7], and the emergence of coacervate droplets as a membrane-free compartmentalization paradigm [8]. International consortia including MaxSynBio, BrisSynBio, BaSyC, fabriCELL, and Build-a-Cell have coordinated large-scale efforts toward synthetic cell construction [9, 10], and a roadmap toward the synthesis of life has recently been proposed [11]. Nevertheless, the integration of individually functional modules into a coherent synthetic system remains elusive [11, 12], and the philosophical question of what constitutes an artificial cell continues to animate productive debate [13].

The bottom-up approach offers a perspective that is particularly relevant to the microbiology and biotechnology community. Rather than studying living microbial cells in their full complexity, bottom-up synthetic biology reconstructs cell-like systems from their most fundamental molecular components, asking which minimal set of biochemical elements is sufficient to reconstitute a given cellular function. This reductionist strategy provides a powerful tool for interrogating the origins and functional logic of microbial life: by assembling simplified analogs of bacterial metabolism, membrane biogenesis, or gene expression from scratch, researchers can identify the core principles governing these processes in ways that are difficult to achieve through the study of intact living cells. In this sense, bottom-up synthetic biology does not replace microbiology but rather offers it a new experimental lens that builds understanding of cellular life from the ground up.

This review provides a comprehensive account of bottom-up approaches to artificial cell design, covering scaffold materials, four canonical functional categories, applications, and the technical challenges that must be addressed to advance the field toward practical realization.

## Key Features of Living Cells

Living cells exhibit a remarkable set of features that collectively define their identity as the basic unit of life. These features are not merely descriptive attributes but represent functional imperatives: each one contributes to the cell's ability to survive, reproduce, and adapt. From the perspective of bottom-up synthetic biology, understanding these features is a prerequisite for designing artificial cell mimics, as each feature represents a design target that researchers seek to partially or fully reconstitute in a non-living system. In this section, we describe six key features of living cells that are central to the field of artificial cell design: confinement, compartmentalization, metabolism, molecular transport, replication and division, and defense (Fig. 1B).

### Confinement

Perhaps the most fundamental feature of any living cell is its possession of an independent, physically bounded space that is distinct from the surrounding environment. This enclosed interior provides the spatial framework within which all other cellular processes take place. By sequestering biochemical components from the external milieu, the cell minimizes interference and establishes an environment in which biochemical reactions can be precisely regulated and optimized. It is widely held that the appearance of such a confined space was a prerequisite for the emergence of the first proto-cells on Earth, and the Russian biochemist Alexander Ivanovich Oparin famously proposed that organic coacervates, microscale droplets formed by spontaneous phase separation of macromolecules, may have provided the earliest form of cellular enclosure [14].

Beyond its role as a physical boundary, confinement provides a profound kinetic advantage. By restricting biocatalytic molecules to an extremely small volume, the effective concentration of reactive species is dramatically increased, leading to enhanced reaction rates and altered molecular crowding effects [5]. This so-called confinement effect, in which enzymatic activity is markedly enhanced relative to bulk solution, is increasingly recognized as a key reason why cellular life evolved to operate at the microscale. The reconstitution of this feature in artificial cells, typically through the encapsulation of enzymes or nucleic acids within micrometer-scale compartments, therefore captures not only a structural attribute of the cell but also a fundamental thermodynamic and kinetic advantage.

### Compartmentalization

Whereas confinement defines the cell as a whole, compartmentalization refers to the subdivision of the cellular interior into distinct functional spaces. This multi-compartment architecture is a hallmark of eukaryotic cells, which house numerous membrane-bound organelles including the nucleus, mitochondria, endoplasmic reticulum, Golgi apparatus, and lysosomes, each harboring specialized biochemical process. It is widely proposed that eukaryotic compartmentalization arose through an evolutionary process of endosymbiosis, in which independent prokaryotic ancestors progressively merged and integrated their functions over billions of years [15].

The functional significance of compartmentalization lies in its capacity to segregate incompatible biochemical processes and to organize metabolic cascades in a spatially controlled manner. Rather than allowing all cellular reactions to occur simultaneously in a single undifferentiated space, compartmentalization enables the cell to regulate the flow of substrates and products between organelles, thereby constituting an interconnected network of cascade reactions. The central dogma of molecular biology provides a canonical example: DNA transcription occurs within the nucleus, the resulting messenger RNA is translated on ribosomes located in the cytoplasm or on the rough endoplasmic reticulum, and the resulting polypeptides undergo post-translational modification in the Golgi apparatus before being trafficked to their final destinations. Reconstituting this kind of hierarchical, multi-step reaction logic within artificial cell systems is a major ambition in bottom-up synthetic biology.

### Metabolism

The capacity for self-sustaining metabolism is one of the most widely cited criteria by which living matter is distinguished from non-living matter. A living cell continuously transforms molecules, extracting energy and material from its environment to maintain its own structure, synthesize new components, and power its various functions. This metabolic activity is mediated primarily by enzymes, highly specific biocatalysts that accelerate chemical transformations with extraordinary efficiency and selectivity under mild physiological conditions.

A canonical example of cellular metabolism is glycolysis, in which glucose is oxidized through a series of enzymatic steps to yield ATP, the universal energy currency of the cell, alongside the concurrent regeneration of the coenzyme nicotinamide adenine dinucleotide (NAD). The coupling of anabolic and catabolic pathways through shared intermediates such as ATP and NAD underscore the interconnected nature of cellular metabolism. In the context of artificial cell design, the reconstitution of metabolic pathways is significant for two reasons. First, it represents a step toward energy self-sufficiency in synthetic systems, reducing or eliminating the need for external energy inputs. Second, it provides a model for studying how simple chemical networks can give rise to the emergent behavior associated with life. Implementing even a subset of these metabolic functions within an artificial cell represents a considerable engineering challenge, requiring the coordinated encapsulation and activity of multiple enzymes within a confined space.

### Molecular Transport

A living cell, while maintaining a chemically distinct interior, is not a closed system. Selective exchange of molecules between the cell and its environment is essential for nutrient uptake, waste excretion, signal reception, and the maintenance of ionic gradients. This transport function is mediated by the cell membrane, which acts as a selectively permeable barrier. Passive mechanisms include simple diffusion, governed by the lipid solubility and molecular size of the permeating species, and facilitated diffusion through membrane channels and carrier proteins. Active transport mechanisms additionally allow the cell to move molecules against their concentration gradient, powered by ATP hydrolysis.

In general, the macromolecular components that constitute the cell are too large to pass through the membrane without assistance, whereas smaller substrate molecules and signaling compounds can diffuse or be transported selectively. This size- and polarity-based selectivity is fundamental to the cell's ability to sustain metabolism by importing nutrients and exporting products, and to participate in intercellular signaling. For the designers of artificial cells, recapitulating selective permeability is a non-trivial challenge, since the ideal scaffold must simultaneously retain encapsulated macromolecular components while permitting the exchange of small molecules necessary for metabolic function.

### Replication and Division

A defining feature of life is the capacity for self-reproduction. In natural cells, cell division is preceded by faithful replication of the genome, ensuring that each daughter cell receives a complete and accurate copy of the genetic information present in the mother cell. The resulting daughter cells are genetically identical to their parent and are capable of independent growth and further division. Self-replication of an information-carrying molecule is widely considered to be a foundational event in the emergence of life, and its reconstitution in artificial systems remains one of the most challenging frontiers in synthetic biology.

Cell growth, which is closely coupled to division, involves the increase in both the mass and volume of the cell. In natural cells, membrane growth is achieved through the de novo synthesis and insertion of phospholipid molecules, a process that expands the membrane surface area in coordination with the enlargement of cellular contents. Following division, the resulting daughter cells are smaller than the original mother cell and must undergo growth before they are capable of further division. The engineering of growth and division in artificial cells is not only of fundamental scientific interest but also has practical implications for the development of self-renewing synthetic systems capable of long-term operational stability.

### Defense

Living cells are composed primarily of biochemical molecules including lipids (approximately 13% of dry cell mass) and proteins (approximately 15%), which are inherently vulnerable to oxidative damage. Reactive oxygen species, including hydroxyl radicals, superoxide anions, and singlet oxygen, can oxidize membrane lipids, denature proteins, and introduce mutations into nucleic acids, all of which are potentially lethal to the cell. In addition, cells face threats from xenobiotics such as viruses and invading bacteria. To counter these threats, cells have evolved elaborate defense mechanisms. A prominent example is the glutathione system, in which the tripeptide glutathione acts as a sacrificial reductant that intercepts oxidative species before they can damage cellular biomolecules. Phagocytosis and lysosomal degradation provide a complementary defense against invading pathogens.

In the context of artificial cell design, the incorporation of antioxidant defense mechanisms is particularly relevant when oxidation-sensitive components such as lipid membranes, enzymes, or nucleic acids are employed, and represents a design target that must be explicitly addressed when photocatalytic modules are integrated into the system, as discussed in Section 4.4.

## Scaffold Materials for Artificial Cells

**Fig. 2:**
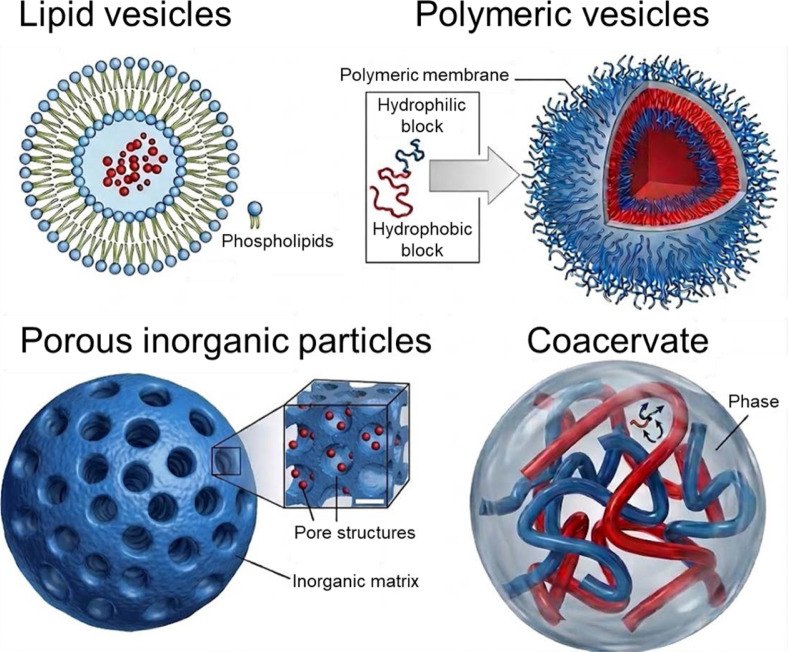
Principal scaffold materials employed in bottom-up artificial cell design: lipid vesicles, polymeric vesicles, porous inorganic particles, and coacervate-based compartments, each offering distinct physicochemical properties and functional trade-offs.

The structural scaffold of an artificial cell defines its physical boundary, determines its permeability to molecular cargo, and sets the physicochemical conditions within which encapsulated biochemical reactions take place. Unlike living cells, which universally employ a phospholipid bilayer as their primary boundary material, artificial cells are not constrained to any single architecture. We introduce five principal scaffold categories employed in artificial cell design: lipid vesicles, polymeric vesicles, porous inorganic particles, coacervate-based compartments, and hybrid hierarchical systems (Fig. 2 and Table 1).

### Lipid Vesicles

Phospholipid bilayers are the most biologically faithful scaffold material for artificial cell construction, sharing both the chemical composition and lamellar architecture of natural cell membranes. The self-assembly of phospholipids into closed bilayer structures is governed by the packing parameter, and structures with a value close to unity spontaneously form bilayer vesicles under aqueous conditions. Because the full phospholipidome of natural cells comprises over a hundred distinct species [16], artificial cell systems are typically assembled from a minimal combination of one to two PC species, one to two PE species, and cholesterol, using palmitoyl (16:0) and oleoyl (18:1) acyl chains as the most common building blocks [17]. The principal limitations of lipid-based scaffolds are their susceptibility to oxidative degradation, disruption by detergents and organic solvents, and restricted permeability. Despite these drawbacks, lipid vesicles remain an indispensable platform owing to their biological relevance.

### Polymeric Vesicles (Polymersomes)

Polymeric vesicles, or polymersomes, are structural analogs of liposomes assembled from amphiphilic block copolymers, with hydrophilic segments typically composed of PEG and hydrophobic segments of PB, PS, PCL, or PDMS [18]. Compared to lipid vesicles, polymersomes offer greater mechanical stability and chemical resistance, and their synthetic versatility allows the incorporation of stimuli-responsive segments that confer sensitivity to temperature, pH, redox potential, or light. Giant polymersomes fabricated in the 1-500 μm diameter range are the most widely employed scaffold format in artificial cell research. Proteinosomes assembled from protein-polymer conjugates such as BSA-PNIPAAm represent a notable variant, offering thermoresponsive permeability and bioconjugation-based crosslinking [19]. A practical limitation is the polydispersity inherent to synthetic polymer chains, which introduces variability in membrane properties, and the non-biodegradable nature of many commonly used polymers limits their suitability for biomedical applications.

### Porous Inorganic Particles

Porous inorganic particles, most notably mesoporous silica, provide compartmentalization through size-exclusion molecular sieving rather than a bilayer membrane [20]. Pore sizes in the mesoporous regime of 2–50 nm allow the free diffusion of small substrates and cofactors with molecular weights below approximately 1,000 Da, while retaining encapsulated enzymes within the particle interior. Silica-based scaffolds offer substantially superior physicochemical stability compared to vesicular systems, being resistant to elevated temperatures, surfactants, hydrolytic degradation, and mechanical stress [20]. Their principal conceptual limitation is that the rigid inorganic shell bears little resemblance to the dynamic fluid membrane of a living cell, and whether such systems qualify as true artificial cells remains debated. Nevertheless, porous silica particles have proven powerful platforms for multi-enzyme cascade reactions, as discussed in Section 4.

### Coacervate-Based Compartments

A qualitatively distinct scaffolding paradigm has emerged from the recognition that not all biological compartments are membrane-bound. Intracellular membraneless organelles such as nucleoli, P-bodies, and stress granules form through liquid-liquid phase separation (LLPS) of proteins and nucleic acids [21], inspiring a new class of membrane-free artificial cell scaffolds. Coacervate microdroplets formed by associative LLPS of oppositely charged macromolecules produce a molecularly crowded interior that markedly enhances encapsulated enzyme activity and nucleic acid condensation [8]. Their dynamic and reversible nature allows assembly and disassembly in response to pH, ionic strength, temperature, or light, enabling stimuli-responsive artificial cell systems. Recent work has demonstrated the spontaneous formation of hierarchically structured core-shell coacervate compartments driven by evaporation-induced phase separation [22]. A significant limitation of bare coacervate droplets is their colloidal instability arising from ultralow interfacial tension, which drives coalescence over time and motivates the development of membranized systems described below.

### Hybrid and Hierarchical Compartment Systems

Recent research has sought to combine the above scaffold types into hybrid architectures that capture the advantages of multiple platforms, motivated by the need to recapitulate the hierarchical compartmentalization of eukaryotic cells. One prominent strategy involves membranization of coacervate microdroplets, in which a stabilizing shell of lipids, block copolymers, protein-polymer conjugates, or polysaccharide layers is assembled at the coacervate-water interface, simultaneously stabilizing the droplet and enabling gated molecular transport [22, 23]. Mann and colleagues demonstrated an extreme case of this approach by assembling living bacteria as building blocks for coacervate membranization, producing a bacteriogenic hybrid protocell of remarkable structural complexity [24]. A complementary approach encapsulates coacervate droplets within pre-formed giant vesicles as artificial membraneless organelles, closely mirroring eukaryotic cell organization [25]. Such systems support spatially segregated enzymatic acceleration and signal-responsive behavior, enabling the implementation of Boolean logic operations within a single protocell [26]. These hybrid and hierarchical systems currently represent the most structurally realistic approximation of cellular organization available to the field.

## Recent Research in Artificial Cell Design

The design of artificial cells from the bottom up encompasses a broad range of experimental strategies, each targeting one or more of the key cellular features described in Section 2. In this section, we review recent research organized around four principal functional categories: cascade metabolism, protein synthesis, division, and energy production. For each category, we first discuss the foundational studies that established key design principles, and then highlight recent advances that have extended the complexity and capability of these systems.

### Cascade Reactions or Metabolism

The reconstitution of multi-enzyme cascade reactions within confined compartments is one of the most widely studied aspects of artificial cell design, as it directly addresses the metabolic function of cells and provides a tractable experimental model for studying the effects of confinement on reaction efficiency. In living cells, metabolic cascades are spatially organized across multiple organelles, with substrates and products transported between compartments. Recreating this spatial logic in an artificial system requires not only the co-encapsulation of multiple enzymes but also the engineering of selective transport pathways between sub-compartments, making cascade metabolism a demanding yet highly informative design challenge.

A foundational study by Elani, Ces, and colleagues at Imperial College London demonstrated spatially segregated cascade reactions within lipid vesicle-based artificial cells [27]. Using unilamellar liposomes subdivided into multiple internal compartments connected by alpha-hemolysin pores of 1.5 nm diameter, they distributed three sequentially acting enzymes across separate sub-compartments: lactase, glucose oxidase, and peroxidase. Substrates diffused through the pores to trigger a stepwise cascade that ultimately produced the fluorescent product resorufin, demonstrating that spatially organized biochemical logic can be implemented within a purely synthetic compartment system.

**Fig. 3:**
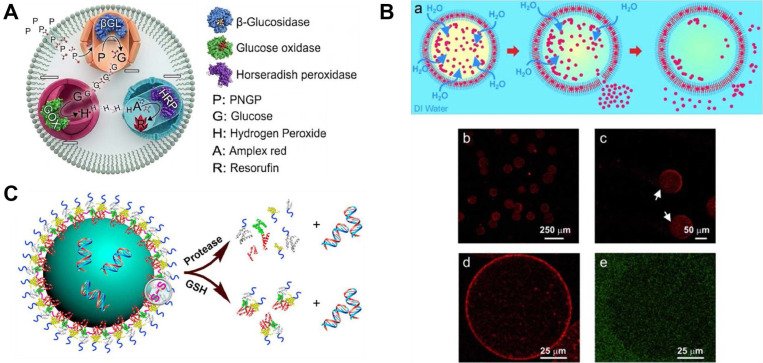
(**A**) Silica nanoreactor-based model artificial cell in which three sequentially acting enzymes are independently encapsulated within separate nanoparticles and combined within a single cell-sized emulsion droplet to reconstitute a multi-step cascade reaction [28]. (**B**) Cell-free gene expression within microfluidically fabricated PEG-b-PLA polymersomes. An *E. coli*-derived transcription-translation system encapsulated within the polymersome lumen successfully synthesized red fluorescent protein, with osmotic shock enabling controlled protein release and membrane self-sealing reproduced with permission [31]. Copyright 2012, Wiley-VCH. (**C**) BSA-PNIPAAm proteinosome-based artificial cell supporting cell-free eGFP synthesis. Thermoresponsive and glutathione-responsive permeability of the protein-polymer shell enables multi-stimulus control over intracellular protein retention and triggered release reproduced with permission [30]. Copyright 2014, American Chemical Society.

Jo, Wurm, and Landfester developed a complementary approach using silica nanoreactor sub-compartments housed within a water-in-oil emulsion as the outer confinement [28] (Fig. 3A). Three enzymes, beta-glucosidase, glucose oxidase, and horseradish peroxidase, were independently encapsulated within separate silica nanoparticles and combined within a single emulsion droplet of 0.5 to 15 μm diameter, comparable in volume to a single cell. The cascade reaction from polysaccharide substrate to resorufin was observed to proceed significantly faster within these confined volumes than in equivalent bulk reactions, providing direct experimental support for the hypothesis that microscale confinement was a driving force in the evolution of cellular life.

Seo and Lee at POSTECH demonstrated programmable enzymatic reaction networks in microfluidically synthesized polymersomes with tunable membrane permeability [29]. By fabricating polymersomes from block copolymers using droplet microfluidics, they produced artificial cells with precisely controlled permeability to specific substrates. This enabled the design of cascade networks in which the flow of biochemical information between artificial cells, or between separate compartments within a single cell, could be programmed by selecting the appropriate membrane composition. The system demonstrated that the programmability of enzymatic logic in artificial cells could approach the sophistication of natural metabolic regulatory networks.

Building on these results, recent work has extended cascade reaction systems to include coacervate-based sub-compartments as artificial organelles within membrane-enclosed outer compartments. The molecularly crowded interior of coacervate droplets has been shown to enhance enzyme activity substantially compared to dilute solution, and the encapsulation of enzyme-loaded coacervates within polymersomes or proteinosomes enables hierarchical cascade architectures in which each sub-compartment contributes a distinct enzymatic step [30]. Signal-responsive control over coacervate assembly and disassembly further allows cascade reactions to be switched on or off in response to external stimuli, adding a regulatory dimension not available in membrane-based systems.

### Protein Synthesis

The reconstitution of the central dogma of molecular biology within an artificial cell, allowing DNA-encoded information to be transcribed and translated into functional proteins, is widely regarded as a hallmark capability of advanced artificial cell systems. In natural cells, this process is tightly coupled to energy supply, ribosome availability, and the spatiotemporal regulation of gene expression. Achieving comparable functionality in an artificial setting requires the co-encapsulation of a complete transcription-translation machinery alongside sufficient energy substrates within a stable and permeable compartment, presenting one of the most demanding integration challenges in the field.

Martino, Cooper, Weitz, and colleagues demonstrated cell-free gene expression within PEG-b-PLA polymersomes fabricated by microfluidic double emulsion [31] (Fig. 3B). By encapsulating an E. coli-derived cell-free transcription-translation system together with a DNA template encoding red fluorescent protein (RFP), amino acids, coenzymes, and energy-regenerating enzymes within the polymersome lumen, they achieved successful in situ synthesis of RFP. Controlled release of the synthesized protein was accomplished by osmotic shock-induced transient permeabilization of the membrane, followed by spontaneous self-sealing, demonstrating a model of protein secretion.

Mann and colleagues demonstrated an alternative scaffold approach using proteinosomes assembled from BSA-PNIPAAm conjugates [19]. Cell-free gene expression of eGFP was achieved within these protein-polymer hybrid compartments, with thermoresponsive release of the synthesized protein triggered by cooling below the lower critical solution temperature of PNIPAAm at 32°C. The introduction of disulfide crosslinks into the proteinosome shell further enabled glutathione-responsive permeability, demonstrating multi-stimulus control over protein retention and release [30] (Fig. 3C).

A major advance in the field has been the development of self-sustaining protein synthesis systems in which the energy required for translation is generated internally rather than supplied exogenously. Berhanu and colleagues combined a PURE cell-free protein synthesis system with proteoliposomes containing purified ATP synthase and bacteriorhodopsin within a giant unilamellar vesicle [32]. Under light illumination, bacteriorhodopsin drove proton pumping across the inner proteoliposome membrane, generating a proton motive force that powered ATP synthase to produce ATP. The photosynthesized ATP fueled the transcription and translation of bacteriorhodopsin and ATP synthase subunits from encapsulated DNA templates, which were then incorporated into the proteoliposomes to further enhance ATP production, creating a positive feedback loop. This system represents a significant step toward energetically autonomous artificial cells capable of self-sustaining gene expression.

Recent work has further explored the use of liquid-liquid phase separation to spatially regulate cell-free protein synthesis within artificial cells. By triggering coacervate formation inside liposomes in response to enzymatically generated signal molecules, it has been shown that the local crowding and macromolecular concentration within de novo assembled coacervate organelles can markedly enhance transcription and translation efficiency, providing a physicochemical mechanism by which the cell-like regulation of gene expression might be achieved in a synthetic context [33].

### Division

The ability of an artificial cell to divide is one of the most challenging and conceptually significant design objectives in the field, as it addresses the capacity for self-reproduction that is a defining characteristic of living systems. In natural cells, division is a tightly orchestrated process involving genome replication, cytoskeletal reorganization, and coordinated membrane growth and fission. Reconstituting even a simplified analog of this process in an artificial system requires the coupling of a chemical or physical driving force to membrane dynamics in a manner that reliably produces daughter compartments while distributing internal contents between them.

Kurihara, Sugawara, and colleagues at the University of Tokyo demonstrated self-reproduction of giant lipid vesicles coupled to the amplification of encapsulated DNA [34]. Cationic lipid vesicles composed of POPC, POPG, and Rhod-DOPE were loaded with PCR machinery. As the internal DNA amplification reaction proceeded, the accumulation of negatively charged DNA products at the inner leaflet of the positively charged membrane created an electrostatic imbalance that destabilized the membrane and drove vesicle growth and eventual division, producing daughter vesicles containing a portion of the amplified DNA. Although the division mechanism differs mechanistically from that of natural cells, this system provided a proof of concept for chemically driven self-reproduction in a lipid-based artificial cell.

**Fig. 4:**
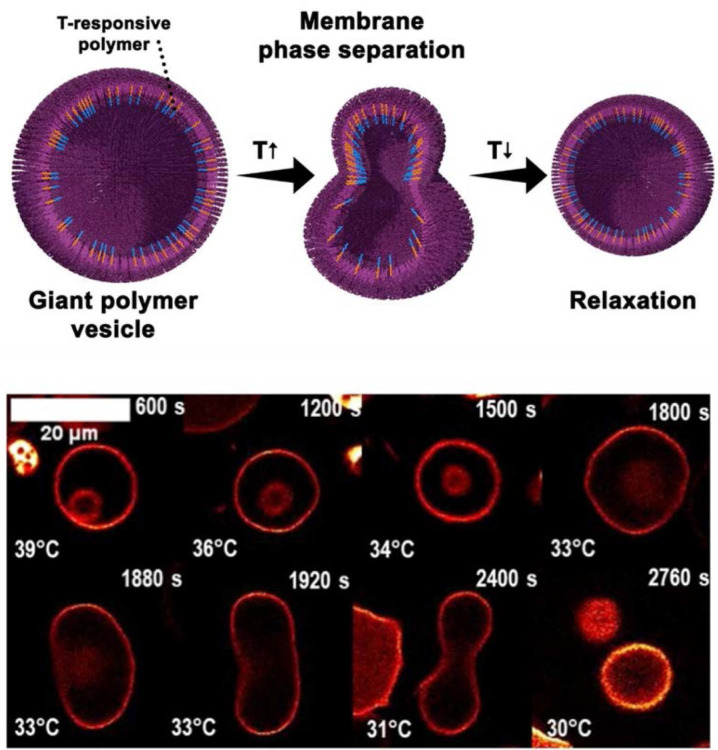
Temperature-triggered division of giant unilamellar polymersomes composed of PDMA-b-PNIPAM and PBD-b-PEO. Cooling below 32°C drives dissociation of the thermoresponsive block from the membrane, generating mechanical perturbations that spontaneously produce daughter polymersomes reproduced with permission [31]. Copyright 2022, Wiley-VCH.

Melchiors, Landfester, and colleagues demonstrated temperature-triggered division of giant unilamellar polymersomes using a binary block copolymer membrane composition [35] (Fig. 4). Polymersomes were fabricated from a mixture of PDMA-b-PNIPAM and PBD-b-PEO in the 200 to 250 μm diameter range. Above 32°C, the thermoresponsive PDMA-b-PNIPAM component integrated into the polymersome membrane. Upon cooling below the lower critical solution temperature, the PNIPAM segments became hydrophilic and dissociated from the membrane, generating mechanical perturbations that drove spontaneous formation of new daughter polymersomes from the parent membrane.

Recent research has addressed a more fundamental challenge: coupling membrane growth to division through in situ lipid synthesis. Phospholipid synthesis within the membrane itself has been demonstrated using reconstituted biosynthetic enzymes encapsulated within liposomes, with the de novo synthesized phospholipids inserting directly into the host membrane and driving an increase in surface area that can ultimately trigger vesicle budding and division [36]. A complementary abiotic approach employed visible light-driven photoredox chemistry to synthesize natural phospholipids from water-soluble precursors in situ, with the generated lipids spontaneously assembling into new vesicle membranes and driving growth and division in a sequence directly analogous to cellular membrane biogenesis [37]. These advances suggest that the coupling of lipid metabolism to membrane dynamics, long recognized as essential in living cells, is now becoming experimentally tractable in artificial cell systems.

### Energy Production

The reconstitution of cellular energy metabolism within artificial cells is essential for achieving the energetic self-sufficiency that would allow synthetic systems to sustain their own biochemical activity without continuous external energy supply. In living cells, ATP is produced through tightly coupled membrane-associated processes, most notably oxidative phosphorylation in mitochondria, which harness electrochemical proton gradients to drive the rotary machinery of ATP synthase. Replicating this proton motive force-driven mechanism within an artificial membrane requires the functional reconstitution of membrane proteins that are notoriously difficult to handle outside their native lipid environment, making energy production one of the most technically demanding frontiers of artificial cell design.

Otrin, Vidakovic-Koch, Sundmacher, Landfester, and colleagues demonstrated the reconstitution of oxidative phosphorylation within polymersomes composed of PDMS-g-PEO [6]. The membrane thickness of approximately 5 nm and its fluid dynamics were matched to the requirements of the membrane proteins bo3 oxidase and F0F1-ATP synthase, both of which were reconstituted into the polymersome membrane. Electron transfer through the oxidase generated a proton gradient across the membrane, which drove ATP synthase to produce ATP from ADP and inorganic phosphate. This artificial mitochondrion system demonstrated that energy transduction machinery, previously studied exclusively in biological membranes, could be functionally reconstituted in a synthetic polymer membrane context.

**Fig. 5:**
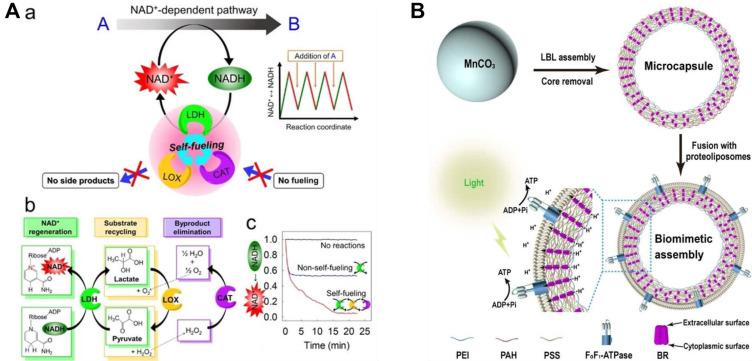
(**A**) Closed-loop NAD regeneration system using silica nanoreactors co-encapsulating LDH, LOX, and CAT. The LDH/LOX/CAT triad continuously interconverts NAD+/NADH while recycling byproducts internally, eliminating the need for external substrate supply or byproduct removal reproduced with permission [38]. Copyright 2021, Wiley-VCH. (**B**) Oriented nanoarchitectonics of bacteriorhodopsin for enhanced light-driven proton pumping and ATP synthesis in a FoF1-ATPase-based reconstituted membrane system, improving the efficiency of proton gradient generation reproduced with permission [40]. Copyright 2022, Wiley-VCH.

NAD regeneration is equally critical for sustaining multi-step metabolic cascades in artificial cells, since NAD-dependent dehydrogenases constitute a large fraction of the enzyme repertoire employed in cell-free metabolic systems. Jo, Wurm, and Landfester demonstrated a closed-loop enzymatic NAD regeneration system using silica nanoreactors co-encapsulating lactate dehydrogenase (LDH), lactate oxidase (LOX), and catalase (CAT) [38] (Fig. 5A). The LDH/LOX/CAT metabolic triad continuously regenerated NAD+ from NADH while simultaneously recycling the lactate byproduct back to pyruvate and detoxifying the hydrogen peroxide byproduct, eliminating the need for continuous substrate supply or byproduct removal. A hybrid photocatalytic system was further developed in which mesoporous core-shell silica nanoreactors combined an encapsulated NAD-converting enzyme with an outer photocatalytic shell for light-driven oxidation of NADH [39]. This system directly confronts the defense challenge described in Section 2.6. The photocatalytic generation of reactive oxygen species poses an oxidative threat to encapsulated enzymes analogous to the ROS-mediated damage that living cells must continuously counteract. The diffusion barrier provided by the silica shell serves as a structural analog of the cellular antioxidant defense system, physically separating the ROS-generating photocatalytic surface from the enzyme interior and thereby protecting catalytic activity over extended operational timescales [39].

Recent research has extended the energy production capabilities of artificial cells by combining ATP synthesis with downstream biochemical functions. Notably, the integration of a light-driven ATP synthesis module with a PURE cell-free protein synthesis system within a single GUV, as described in Section 4.2, demonstrated that photosynthesized ATP could directly power transcription and translation [32]. This coupling of energy generation with protein synthesis represents a step toward artificial cells capable of self-sustaining operation. Further advances have explored the use of quantum dot photosensitizers and oriented bacteriorhodopsin incorporation strategies to improve the efficiency and reproducibility of light-driven proton pumping, which has historically been limited by the random orientation of bacteriorhodopsin molecules within reconstituted membranes [40] (Fig. 5B).

## Applications of Artificial Cells

The functional capabilities demonstrated in Section 4 carry substantial implications for a broad range of applied fields. Artificial cells offer several advantages over living cells as technological tools: they can be fabricated in large quantities under defined conditions, stored without metabolic maintenance, operated in environments hostile to living organisms, and engineered to perform specific biochemical tasks without interference from competing cellular processes. In this section, we discuss four principal application domains: industrial biocatalysis and bioproduction, therapeutic protein delivery, biosensing and diagnostics, and the study of the origins of life.

### Industrial Biocatalysis and Bioproduction

Artificial cells address key limitations of cell-free enzyme cascade systems, namely enzyme instability, cofactor depletion, and catalyst recovery, by providing a confined microenvironment in which enzymes are co-localized, protected from external perturbates, and separated from the bulk phase by a selectively permeable barrier [19]. The self-contained NAD regeneration systems described in Section 4.4, which operate without external substrate replenishment, exemplify this principle [38]. The modular design of artificial cells, in which different catalytic functions are independently encapsulated in distinct sub-compartments and combined on demand, offers a flexible platform for constructing multi-step synthetic pathways that would be difficult to execute in a homogeneous reaction vessel. As fabrication methods mature, artificial cells are increasingly viewed as a viable alternative to whole-cell fermentation for producing fine chemicals, pharmaceutical intermediates, and specialty biomolecules [41, 42].

### Therapeutic Protein Delivery and Drug Factory Applications

Artificial cells equipped with cell-free gene expression machinery offer a qualitatively distinct capability from conventional drug delivery systems: the ability to synthesize therapeutic proteins on demand at the site of action. Krinsky and colleagues demonstrated that vesicle-based artificial cells encapsulating a cell-free expression system could synthesize therapeutic proteins directly within tumor tissue, producing locally high concentrations with reduced systemic exposure [43]. Chen and colleagues extended this concept by implanting artificial cells engineered to produce recombinant growth factors at a tissue graft site in mice, successfully triggering angiogenesis and tissue regeneration without eliciting a systemic immune response over prolonged periods [44]. Strategies inspired by red blood cell biology, including membrane deformability optimization and surface functionalization, have been applied to giant vesicles to extend their circulatory lifetime, advancing their suitability as systemic drug delivery vehicles [45]. The convergence of cell-free protein synthesis with these improved scaffolds is regarded as the foundation for a next generation of programmable therapeutic platforms [46].

### Biosensing and Diagnostics

Artificial cells can be engineered to couple the detection of a specific molecular input to a defined biochemical output, such as the synthesis of a fluorescent reporter protein or the release of a detectable product. The signal amplification afforded by in situ protein synthesis offers an inherent sensitivity advantage over passive binding-based biosensor formats. Integration of cell-free gene expression within vesicle compartments has enabled artificial cells that respond to specific biomarker molecules by producing tunable outputs [47]. Beyond in vitro diagnostics, stimuli-responsive vesicles have been demonstrated as distributed artificial organelles that translate physical inputs into chemical signals recognized by living bacteria, enabling externally controlled activation of gene expression without host genetic modification [48].

### Origins-of-Life Research

Artificial cells serve as powerful experimental models for investigating the minimal chemical conditions necessary for the emergence of cellular life. The confinement effect studies described in Section 4.1 provided experimental support for the hypothesis that microscale confinement was a key driver of early metabolic efficiency [28]. Coacervate droplets, which form spontaneously from mixtures of oppositely charged macromolecules, are studied as models of the earliest proto-cells, and their ability to concentrate RNA and enhance ribozyme activity raises the possibility that LLPS-driven compartmentalization preceded membrane-based enclosure in the history of life [8, 20]. The demonstration that visible-light-driven photoredox chemistry can generate natural phospholipids from water-soluble precursors and drive spontaneous vesicle formation and division further provides a plausible abiotic mechanism for early membrane assembly without biological catalysts [37].

## Technical Limitations and Future Challenges

Despite the remarkable progress surveyed in the preceding sections, bottom-up synthetic biology remains far from its ultimate goal of constructing a fully autonomous artificial cell. Individual functional modules including cascade metabolism, protein synthesis, division, and energy production have each been reconstituted with increasing sophistication, yet their integration into a coherent, self-sustaining system remains elusive. The field now faces a transition from demonstrating isolated functions to assembling them into a unified whole, exposing technical and conceptual challenges that reflect fundamental gaps in our understanding of how cellular subsystems interact and co-depend.

### Module Compatibility and System Integration

The most fundamental challenge facing the field is the integration of individually functional modules into a coherent, co-operating system. Each subsystem reviewed here has been optimized in isolation under conditions that frequently conflict with those required by other modules, as buffer compositions, pH optima, temperature ranges, and cofactor requirements suitable for one subsystem may be inhibitory for another. This incompatibility becomes increasingly severe as more modules are combined, and no artificial cell system to date has successfully integrated all four functional categories within a single compartment [11, 12].

### Stability and Operational Lifetime

The operational lifetime of current artificial cell systems is limited by the chemical instability of their components. Lipid membranes are susceptible to oxidative degradation and hydrolysis, while encapsulated enzymes and cell-free expression systems suffer from progressive inactivation over time. Although polymersomes and silica-based scaffolds offer improved chemical stability, they introduce trade-offs in membrane fluidity and biological compatibility. The development of artificial cell scaffolds that combine long-term physicochemical stability with the dynamic responsiveness required for functional operation remains an open challenge.

### Ethical and Regulatory Considerations

As artificial cells move closer to biomedical application, ethical and regulatory frameworks must evolve in parallel. Regulatory agencies including the FDA have not yet established clear evaluation pathways for artificial cell-based therapeutics, which occupy a conceptual space between conventional drug delivery systems and living cell therapies. Questions of biosafety, including the potential consequences of artificial cell release into biological or ecological environments, require careful prospective analysis. At the same time, the replacement of living cells with artificial systems in biotechnological and research applications offers meaningful ethical advantages by reducing reliance on cell cultures derived from living organisms, a consideration that should be weighed alongside the technical challenges of the field.

## Conclusion

Bottom-up synthetic biology has progressed substantially from its early demonstrations of single-enzyme encapsulation toward the assembly of multi-functional artificial cell systems capable of cascade metabolism, protein synthesis, membrane division, and energy transduction. The expansion of scaffold platforms to include coacervate-based and hybrid hierarchical compartments has broadened the structural vocabulary available to artificial cell designers, while advances in cell-free gene expression and in situ lipid synthesis have brought the field closer to energetically self-sufficient synthetic systems. Applications spanning industrial biocatalysis, therapeutic protein delivery, biosensing, and origins of life research underscore the translational potential of these platforms. Nevertheless, the integration of functional modules into coherent, stable, and scalable systems remains the central unresolved challenge. Addressing this challenge will require sustained interdisciplinary collaboration and the development of standardized design frameworks, but the field is now positioned to deliver transformative advances in both fundamental biology and biotechnology.

## Figures and Tables

**Table 1:** Comparative properties of five principal scaffold materials used in bottom-up artificial cell design.